# Potential biomarkers and therapeutic targets in cervical cancer: Insights from the meta-analysis of transcriptomics data within network biomedicine perspective

**DOI:** 10.1371/journal.pone.0200717

**Published:** 2018-07-18

**Authors:** Medi Kori, Kazim Yalcin Arga

**Affiliations:** Department of Bioengineering, Faculty of Engineering, Marmara University, Istanbul, Turkey; Penn State University School of Medicine, UNITED STATES

## Abstract

The malignant neoplasm of the cervix, cervical cancer, has effects on the reproductive tract. Although infection with oncogenic human papillomavirus is essential for cervical cancer development, it alone is insufficient to explain the development of cervical cancer. Therefore, other risk factors such as host genetic factors should be identified, and their importance in cervical cancer induction should be determined. Although gene expression profiling studies in the last decade have made significant molecular findings about cervical cancer, adequate screening and effective treatment strategies have yet to be achieved. In the current study, meta-analysis was performed on cervical cancer-associated transcriptome data and reporter biomolecules were identified at RNA (mRNA, miRNA), protein (receptor, transcription factor, etc.), and metabolite levels by the integration of gene expression profiles with genome-scale biomolecular networks. This approach revealed already-known biomarkers, tumor suppressors and oncogenes in cervical cancer as well as various receptors (e.g. ephrin receptors EPHA4, EPHA5, and EPHB2; endothelin receptors EDNRA and EDNRB; nuclear receptors NCOA3, NR2C1, and NR2C2), miRNAs (e.g., miR-192-5p, miR-193b-3p, and miR-215-5p), transcription factors (particularly E2F4, ETS1, and CUTL1), other proteins (e.g., KAT2B, PARP1, CDK1, GSK3B, WNK1, and CRYAB), and metabolites (particularly, arachidonic acids) as novel biomarker candidates and potential therapeutic targets. The differential expression profiles of all reporter biomolecules were cross-validated in independent RNA-Seq and miRNA-Seq datasets, and the prognostic power of several reporter biomolecules, including KAT2B, PCNA, CD86, miR-192-5p and miR-215-5p was also demonstrated. In this study, we reported valuable data for further experimental and clinical efforts, because the proposed biomolecules have significant potential as systems biomarkers for screening or therapeutic purposes in cervical carcinoma.

## Introduction

Cervical cancer is a malignant neoplasm originating from cells derived from cervix squamocolumner junction of the uterine cervix. It is the second most common cancer and one of the leading causes of cancer death among women worldwide, especially when the cancer is diagnosed at an advanced stage. Infection by “highly oncogenic” Human Papillomavirus (HPV) is essential for cervical cancer development [1]. More than hundred HPV types have been identified, and these types differ in their oncogenic potential [2–4]. HPV-16 and -18, which are the two HPV s types that are responsible for up to 78% of cervical cancer cases [2]. In addition to these two types, HPV-31, -33, -35, -39, -45, -51, -52, -56, -58 and -59 are defined as high-risk HPV types according to the World Health Organization (WHO) [3]. However, although infection by highly oncogenic HPVs is essential for the development of cervical cancer, it alone is not sufficient; therefore, other cancer related risk factors such as host genetic factors (i.e., gene and chromosome alterations, changes in levels of tumor suppressors and activators) are necessary for this disease to deveop [4, 5]. Therefore, there is an urgent need to clarify the molecular mechanisms behind cervical cancer.

In cases in which the medical diagnosis of cervical cancer is made at a late stage, the mean survival is less than one year [4]; therefore, it is crucial to develop effective screening tests that are capable of providing early detection and prevention. Pap smear is a form of HPV DNA testing that is widely used in screening; however, there are limitations regarding its specificity and sensitivity [6]. Despite the presently available screening tests, nearly 270,000 deaths and 530,000 new cases of cervical cancer occur annually around the world; this finding shows the inadequacy of existing screens and the need for effective screening strategies [7]. Consequently, the elucidation of potential biomarkers for the screening, diagnosis, and monitoring of cervical cancer constitutes a significant research area for further research.

The computational integration of biomolecular networks with data from different omic levels represents the core of research in the field of systems biology. This interdisciplinary field provides valuable information on genome reprogramming under disease conditions and relevant biological entities that might be considered potential diagnostic or therapeutic targets [8]. In this context, considering the unclear etiology of cervical cancer and the inaccuracy of present screening methods, systems-level approaches are needed. Despite individual gene expression studies having explored the mechanisms behind cervical cancer [9–13], systems-level integrative analyses, which predict genes, proteins, and miRNAs as candidate biomarkers or therapeutic targets in this disease, are limited in the literature. For instance, an analysis of promoter sequences of the differentially expressed genes (DEGs) and binding sites of transcription factors (TFs) proposed the TF E2F as a critical transcriptional regulator and a potential molecular target for cervical cancer therapy [14]. In another study, the miRNAs, miR-203 and miR-30b and the target genes *BIRC5*, *HOXA1*, and *RARB* were suggested to be critical players in the pathogenesis of cervical cancer, as revealed by the systematic analysis of dysregulated miRNAs and their targets in cervical cancer [15]. Furthermore, by integrating the human protein interaction data and cervical cancer gene sets, several novel candidates (e.g., VEGFA and IL-6) genes involved in cervical carcinogenesis were also predicted [16, 17].

Although these studies have provided significant findings about cervical cancer, conclusions about the central molecular mechanisms behind the disease were not reached because this type of information requires an integrative multi-omics approach. Considering the intertwined structure of signaling, regulatory and metabolic processes within a cell, we employed three genome-scale biomolecular networks (protein-protein interaction (PPI), metabolic, and post-transcriptional regulatory networks) for the first time in analyzing cervical cancer. Accordingly, a meta-analysis of the cervical cancer associated transcriptomic datasets was performed by taking into consideration five independent studies and a total of 236 samples, and the core information on DEGs was obtained by statistical analyses. Gene set over-representation analyses were performed on core DEGs to identify significantly enriched pathways and Gene Ontology (GO) terms. DEGs were further integrated with genome-scale human biomolecular networks: (i) a PPI network was reconstructed around the core DEGs, and topological analyses were performed to predict hub proteins that play central roles in signal transduction and reporter receptors; (ii) reporter metabolites were revealed by using the genome-scale human metabolic model; and (iii) reporter TFs and miRNAs were proposed by the reconstruction of a transcriptional and post-transcriptional regulatory network (incorporating miRNA-target gene and TF-target gene interactions). Moreover, survival analyses were performed and the prognostic power of selected reporter biomolecules was identified via cross-validation using independent gene expression datasets. Consequently, this systematic study reports candidate biomolecules that can be considered as diagnostic/prognostic biomarkers or potential therapeutic targets for further experimental and clinical trials for cervical cancer.

## Materials and methods

### Gene expression datasets of cervical cancer

To analyze the gene expression profiles in cervical cancer, five independent transcriptome datasets (GSE7803, GSE9750, GSE39001, GSE52903, and GSE63514) [9–13] including data from cervical epithelium samples were obtained from the Gene Expression Omnibus (GEO) database [18]. To avoid undesirable alterations originating from differences in the microarray platforms used, only Affymetrix microarrays (i.e., Human Genome U133 Plus 2.0, Human Gene 1.0 ST, and Human Genome U133A) were employed. Furthermore, samples from cervical intraepithelial neoplasm were excluded from the GSE63514 and GSE7803 datasets to prevent sample heterogeneity. The final number of samples considered in transcriptome datasets with their HPV profiling is given in Table 1. In addition to eight specimens with unknown HPV profiles in GSE63514 and two HPV-negative samples in GSE9750, all HPV types associated with diseased specimens belonged to highly carcinogenic HPV types, in accordance with the WHO classification. Consequently, a total of 156 cervical cancer samples and 80 healthy samples were examined.

**Table 1:** Transcriptome datasets employed in the present study.

GEO ID	# of Tumor Samples	HPV type(s): # of samples	# of Control Samples	Reference Study
GSE7803	21	HPV16: 10, HPV18: 4, HPV18/45: 1, HPV33/52/58: 4, HPV58: 1, HPV59: 1	10	[9]
GSE9750	33	HPV16: 19, HPV18: 3, HPV45: 4, HPV16/18: 1, HPV18/45: 1, HPV16/31/45: 2, HPV16/18/31/45: 1, HPV (-): 2	24	[10]
GSE39001	19	HPV16: 19	5	[11]
GSE52903	55	HPV16: 55	17	[12]
GSE63514	28	HPV16: 19, HPV18: 1, Unspecified: 8	24	[13]

### Identification of differentially expressed genes

A previously constructed statistical analysis procedure [19] was adopted in the present study to determine the DEGs. In summary, the raw data (stored in. CEL files) of each dataset were normalized by calculating the Robust Multi-Array Average (RMA) expression measure (version 1.30.1) [20] as implemented in the Affy package (version 1.56.0) [21] of R/Bioconductor platform (version 3.3.0) [22]. DEGs were identified from the normalized expression values by using the Linear Models for Microarray Data (LIMMA) package (version 3.34.5) [23]. The Benjamini-Hochberg method was used to control the false discovery rate. To determine the statistical significance, adjusted p < 0.01 was used. The regulatory pattern of each DEG (i.e., down- or up-regulation) was determined by fold changes, and at least a 50% change was accepted as significant. Further analyses were performed with the mutual DEGs among all five datasets, so-called “the core genes of cervical cancer”. The descriptions of gene products were obtained from GeneCards: The Human Gene Database [24].

### Gene set overrepresentation analyses

Overrepresentation analyses were performed using the DAVID bioinformatics tool (version 6.8) [25] to identify functional annotations (i.e., biological processes, molecular functions, signaling and metabolic pathways, and diseases) significantly associated with the core genes of cervical cancer. In the analyses, the Kyoto Encyclopedia of Genes and Genomes (KEGG) [26] and Genetic Association Database (GAD) [27] were preferably used as the pathway and disease databases, respectively. Gene Ontology (GO) terminology [28] was employed as the source for annotating the molecular functions and biological processes. P-values were obtained via Fisher’s Exact Test. Benjamini-Hochberg’s correction was used as the multiple testing correction technique, and gene set enrichment results with adjusted p < 0.05 were considered statistically significant.

### Reconstruction of protein-protein interaction networks and topological analysis

The physical interactions of the proteins encoded by the core genes of cervical cancer were analyzed by the reconstruction of PPI networks. For this purpose, the high confidence human protein interactome [29] (with confidence score ≥ 0.8) was employed. PPI networks were reconstructed around down- and up-regulated genes separately and were represented as undirected graphs (i.e., proteins as nodes, and interactions between proteins as edges) in Cytoscape (v3.5.0) [30]. To determine hub proteins, topological analyses were performed using the Cytohubba plugin [31]. A dual-metric approach [32] that simultaneously utilizes a local metric (i.e., node degree) and a global metric (i.e., betweenness centrality) was employed. The degree of a node describes the number of edges of that node, and the betweenness centrality metric defines the number of times a node acts as a bridge along the shortest paths between any two other nodes. The top 5% of proteins with the highest degree and/or betweenness centrality metrics in the PPI network were considered hub proteins.

### Identification of reporter metabolites associated with cervical cancer

To identify reporter metabolites around which significant transcriptional changes occur, the statistically significant changes in gene expression profiles were mapped onto the Human Metabolic Reaction (HMR 2.0) model [33] through the reporter metabolites algorithm implemented in the BioMet Toolbox (v2.0) [34]. The p-values representing the significance of metabolites were corrected by Benjamini-Hochberg’s method and reporter metabolites with an adjusted p-value of < 0.05 were considered statistically significant. The overrepresentation of reporter metabolites in metabolic pathways was determined using the pathway annotations presented by the Metabolites Biological Role (MBRole) database (v2.0) [35].

### Identification of reporter receptors, transcription factors and miRNAs

The reporter features algorithm [36] was adapted and implemented in MATLAB (R2010) to identify reporter receptors, TFs, and miRNAs. The original algorithm was integrated differential transcription data (in terms of p-values representing the significance of gene expression changes) with a metabolic model (consisting of gene-reaction-metabolite associations) to identify reporter metabolites. Each metabolite in the model was scored according to the transcriptional changes in its adjacent genes, which encode enzymes catalyzing the metabolic reactions associated with that metabolite. The metabolites with the highest scores were defined as reporters [36]. Previously, we adapted this algorithm to identify reporter TFs and miRNAs by integrating differential transcription data with the transcriptional and post-transcriptional regulatory network (representing TF-target gene, and miRNA-target gene interactions) to identify reporter TFs and miRNAs [19, 37–39]. To generate a TF-target gene network, experimentally validated TF-target gene interactions were obtained from the combinatorial human transcriptional regulatory interaction network [40] and Human Transcriptional Regulation Interactions database (HTRIdb) [41]. Similarly, to generate miRNA-target gene network, the experimentally validated miRNA-target gene interactions were obtained from our previous study [40] and from miRTarbase (release 6.0) [42].

In this study, we adapted the algorithm to identify reporter receptors by using a receptor-protein interaction network. For this purpose, the proteins that have receptor activity (GO: 0004872) were screened in DAVID [25], PANTHER [43], and Genecodis [44] databases, and the physical interactions of these receptors were extracted from the human protein interactome [29]. By following the same procedure as that in the original algorithm, the p-values representing the significance of gene expression changes in cervical cancer were converted to z-scores by using inverse cumulative distribution and integrated with the receptor-protein interaction network to assign a score to each receptor on the basis of the z-scores of its adjacent proteins. Thereafter, the scores following a standard normal distribution were converted to p-values, and statistically significant (p < 0.05) receptors were assigned as reporter receptors.

### Cross-validation of the reporter biomolecules

The prognostic power of reporter biomolecules (i.e., 10 hubs, 18 receptors, 3 TFs, and 16 miRNAs) was analyzed at the transcriptome level by using independent gene expression (RNA-Seq or miRNA-Seq) datasets obtained from The Cancer Genome Atlas (TGCA). The RNA-Seq dataset consists of 191 samples with their clinical information. The subjects were partitioned into low- and high-risk groups according to their prognostic index, and survival multivariate analyses and risk assessments were performed by SurvExpress [45]. For reporter miRNAs, the cervical cancer associated miRNA-Seq data from TCGA, which consist of 289 patients, were employed to classify the patients into high- and low-risk groups by using OncomiR [46]. The differences in gene expression levels between the risk groups were represented via box-plots, and the statistical significance of the differences was estimated by t-test. The survival signatures of reporter biomolecules were evaluated by Kaplan-Meier plots, and a log-rank p-value < 0.05 was considered the cut-off to describe statistical significance in all survival analyses.

## Results

### The transcriptomic codes of cervical cancer

**Fig 1:**
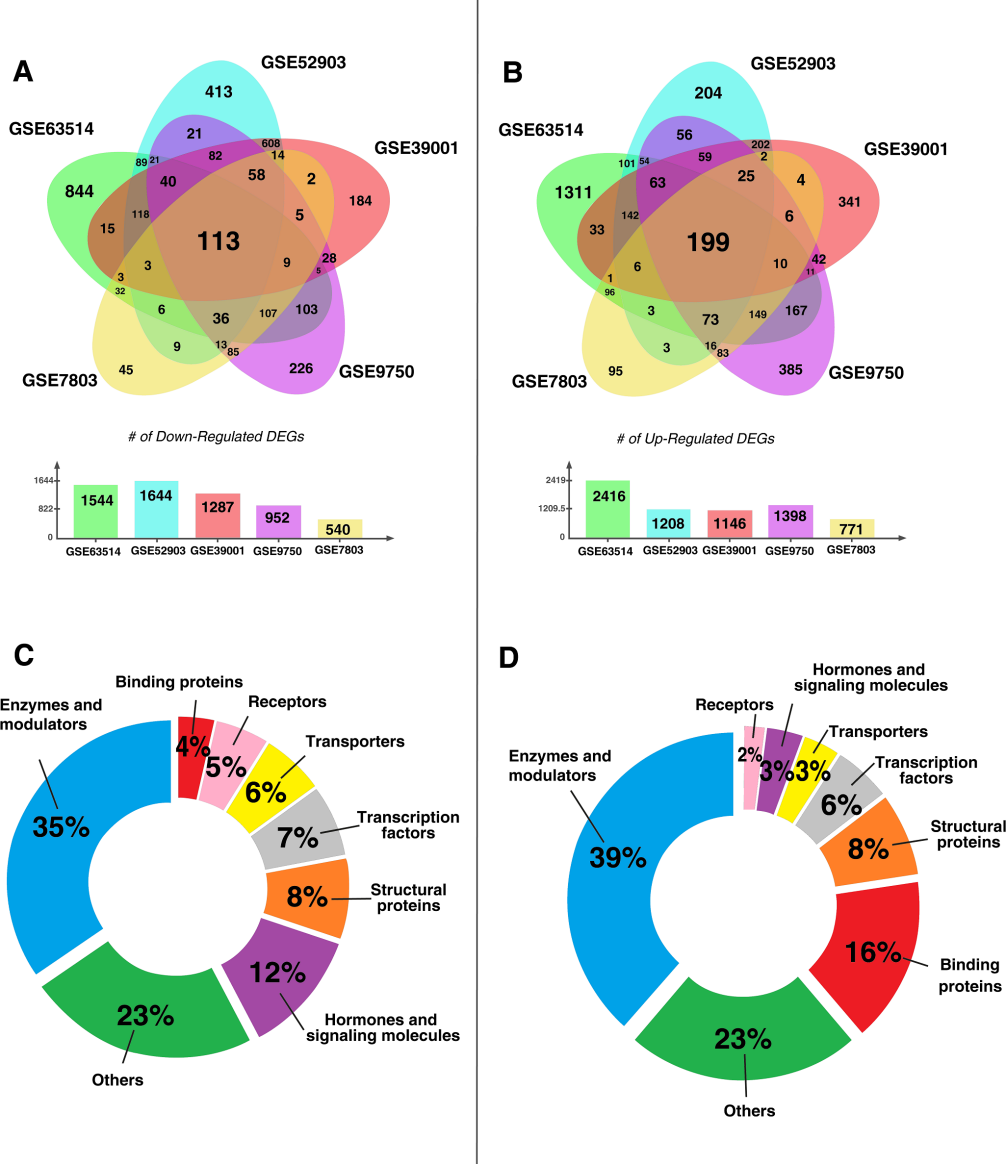
Meta-analysis of the transcriptome datasets associated with cervical cancer. (A) Venn diagram representing the distribution of the down-regulated transcripts in the datasets, where 113 transcripts were mutually down-regulated in all datasets (i.e., down-regulated core genes). (B) Venn diagram representing the distribution of the up-regulated transcripts in datasets, where 199 transcripts were mutually up-regulated in all datasets (i.e., up-regulated core genes). (C) The clustering of the proteins encoded by the down-regulated core genes of cervical cancer according to their molecular activities. (D) The clustering of the proteins encoded by the up-regulated core genes of cervical cancer according to their molecular activities (DEGs: differentially expressed genes).The gene set overrepresentation analysis of the core genes based on the annotations stored in KEGG and GAD databases resulted in (particularly cancers), p53 signaling, and pyrimidine metabolism (Fig 2). Periodontitis, hypospadias, and arterial blood pressure pathways were down-regulated, whereas up-regulated core genes were enriched in those associated with the cell cycle, DNA replication, oocyte meiosis, several cancers (colorectal, bladder, breast, ovarian, lung, stomach, and prostate), autoimmune disorders (including rheumatoid arthritis and systemic lupus erythematosus), Alzheimer's disease, p53 signaling pathway, and pyrimidine metabolism.

The individual statistical analyses of five gene expression datasets resulted in the identification of hundreds of up- and down-regulated DEGs (Fig 1A and 1B) and revealed the reprogramming of 3%-10% of the genome in cervical cancer. The comparative analysis indicated down-regulation of 113 genes and up-regulation of 199 genes in all five transcriptome datasets analyzed in this study (S1 Table). These genes are the so-called “core genes” of cervical cancer. The regulatory patterns of all core genes were consistent among datasets, except for *EGR1*, which was up-regulated in four of the five datasets.

**Fig 2:**
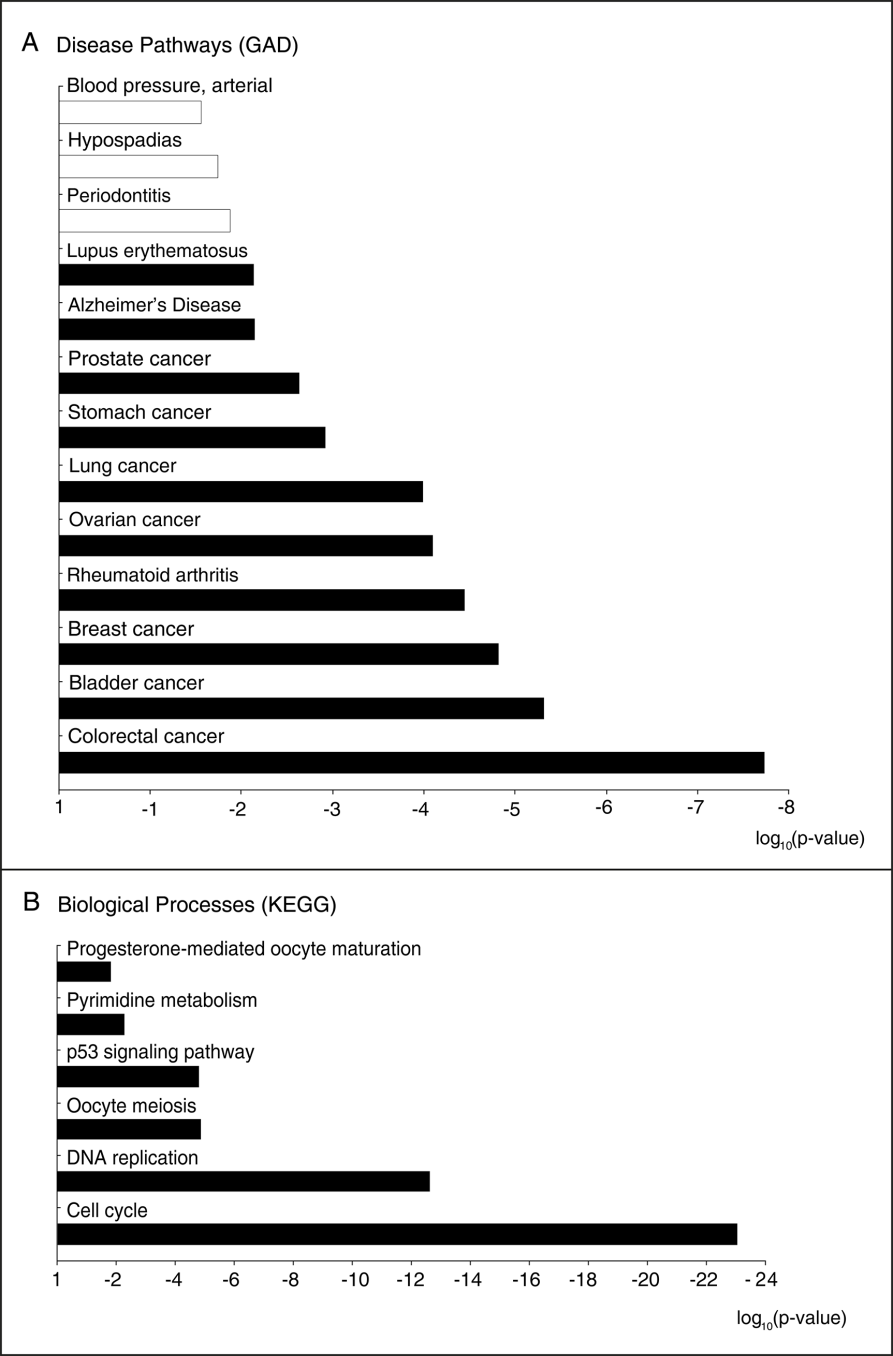
Gene set enrichment analysis of the core genes of cervical cancer. (A) Significantly enriched disease pathways based on the gene-disease associations presented by the Genetic Association Database (GAD). (B) Significantly enriched biological processes based on the gene-process associations of the Kyoto Encyclopedia of Genes and Genome (KEGG) database. The white bar represents down-regulation of the pathway or process, whereas the black bars represents up-regulation.

The core genes of cervical cancer were classified into diverse groups according to their functions and activities. Proteins encoded by the down-regulated core genes mainly comprised enzymes and modulators (35%), hormones and signaling molecules (12%), structural proteins (8%), TFs (7%), transporters (6%), receptors (5%) and binding proteins (4%) (Fig 1C), whereas up-regulated core genes encoded enzymes and modulators (39%), proteins with binding activity (16%), structural proteins (8%), TFs (6%), transporters (3%), hormones and signaling molecules (3%) and receptors (2%) (Fig 1D). Overall, 23% of the core genes of cervical cancer encoded proteins with either miscellaneous functions or unknown functional activities.

### The proteomic codes of cervical cancer

**Fig 3:**
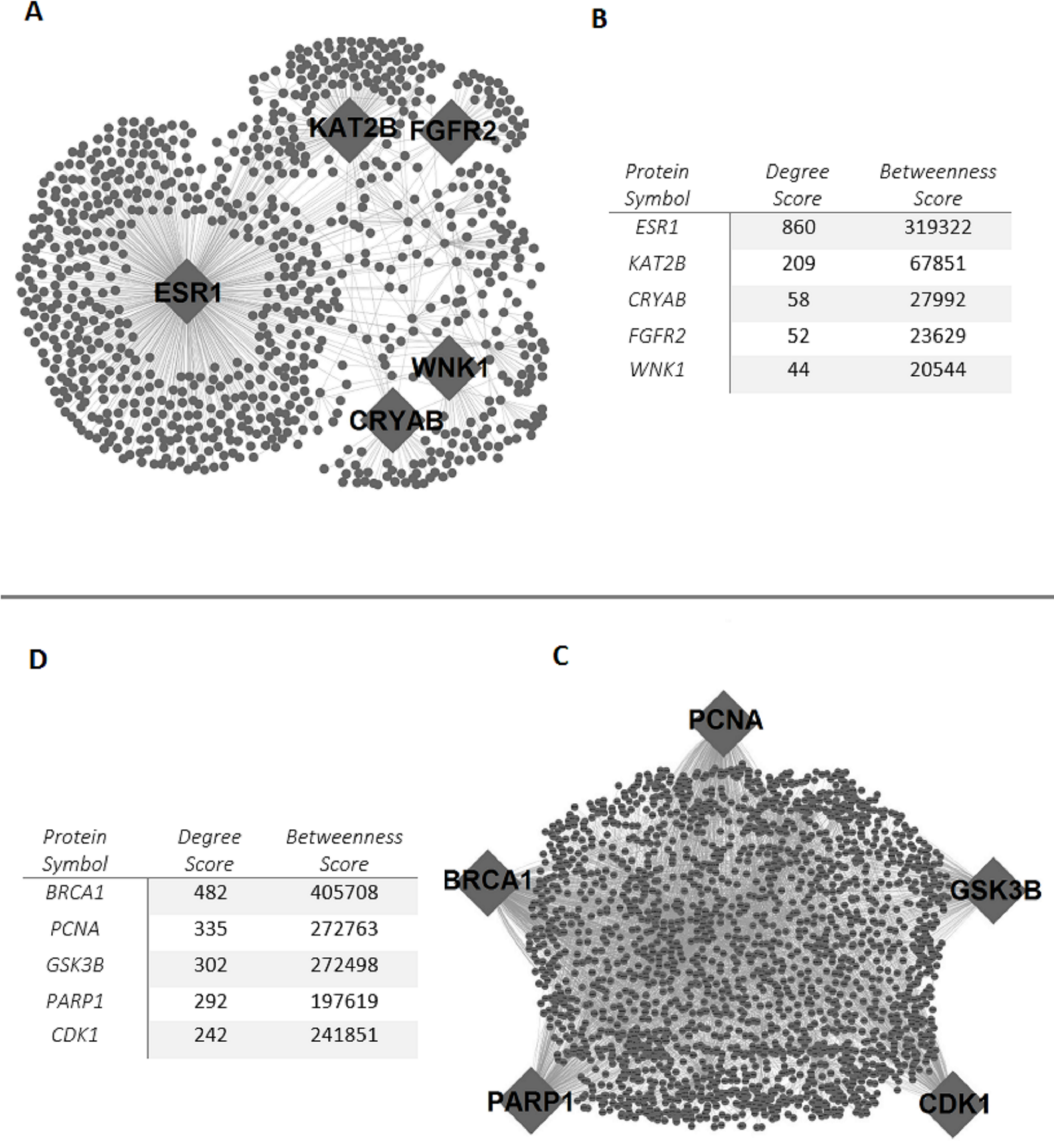
Protein-protein interaction (PPI) sub-networks in cervical cancer. (A) PPI sub-network around the proteins encoded by the down-regulated core genes. (B) PPI sub-network around the proteins encoded by the up-regulated core genes. (C) Hub proteins of the down-regulated PPI sub-network and their topological metrics. (D) Hub proteins of the up-regulated PPI sub-network and their topological metrics.

PPI sub-networks were reconstructed around proteins encoded by the core genes of cervical cancer (Fig 3). The down-regulated PPI sub-network consisted of 907 proteins (i.e., 113 down-regulated core proteins and their physically interacting first neighbors) and 1549 links (i.e., physical PPIs between these proteins), whereas the up-regulated PPI sub-network was composed of 6321 links around 2133 proteins (199 up-regulated core proteins and their interacting first neighbors). Hub proteins that play central roles in modular organization and information flow within the network were identified by the topological analysis of the reconstructed sub-networks. The hub proteins of the down-regulated PPI network were the estrogen receptor (ESR1), lysine acetyltransferase enzyme (KAT2B), crystalline heat shock protein (CRYAB), fibroblast growth factor receptor (FGFR2), and the serine/threonine protein kinase (WNK1) (Fig 3C). The enzymes breast cancer type 1 susceptibility protein (BRCA1), proliferating cell nuclear antigen (PCNA), poly(ADP-Ribose) polymerase 1(PARP1), and the serine/threonine protein kinases; glycogen synthase kinase 3 beta (GSK3B) and cyclin dependent kinase 1 (CDK1) were the up-regulated hub proteins (Fig 3D).

### The metabolic codes of cervical cancer

The reporter metabolites were identified by the integration of transcriptome data with the genome-scale human metabolic network (HMR 2.0). To obtain more insight into metabolic activities around reporter metabolites, pathway enrichment analyses were also performed (Table 2). The most significant pathway was arachidonic acid metabolism (p-value < 10^−6^), which was associated with 15 reporter metabolites such as several derivatives of eicosatetraenoic acid, leukotrienes (A4, B4), arachidonate, 5,6-epoxytetraene and hepoxilin A3. Furthermore, peroxisome proliferator-activated receptor (PPAR) signaling (p-value = 4.22×10^−6^), glutathione metabolism (p-value = 1.83×10^−4^), linoleic acid metabolism (p-value = 9.83×10^−4^), and glycolysis (p-value = 2.38×10^−2^) were also significantly enriched with the reporter metabolites.

**Table 2:** Significantly enriched metabolic pathways in cervical cancer and associated reporter metabolites.

**Metabolic Pathway**	**p-value**	**Reporter Metabolites Enriched In the Metabolic Pathway**
Arachidonic acid metabolism	<10^−15^	5(S)-HETE, 8(S)-HETE, 12(S)-HETE,15(S)-HETE, 5(S)-HPETE, 8(S)-HPETE, (11R)-HPETE, 12(S)-HPETE, 15(S)-HPETE, leukotriene A4, leukotriene B4, arachidonate, 5-oxo-ETE, 5,6-epoxytetraene and hepoxilin A3
Peroxisome proliferator-activated receptor (PPAR) signaling pathway	4.22×10^−6^	8(S)-HETE, 13(S)-HODE and leukotriene B4
Glutathione metabolism	1.83×10^−4^	GSH, GSSG, NADPH and NADP+
Linoleic acid metabolism	9.83×10^−4^	Arachidonate, 13(S)-HODE and 13(S)-HPODE
Glycolysis / Gluconeogenesis	2.38×10^−2^	3-phospho-D-glycerate and 1,3-bisphospho-D-glycerate

### The receptor codes of cervical cancer

To the best of our knowledge, the reporter receptors of cervical cancer were determined for the first time in this study. The significance of receptors was determined by the differential expression patterns of their physically interacting partners. The results show that 18 proteins were identified as reporter receptors with a significance level of p-value <0.005 (Table 3). Among these 18 receptors, two endothelin receptors (EDNRA and EDNRB), three ephrin receptors (EPHA4, EPHA5 and EPHB2), and three nuclear receptors (NCOA3, NR2C1, and NR2C2) were included. Furthermore, ATR (p-value = 8.88×10^−16^), P2RX4 (p-value = 6.05×10^−12^), EGFR (p-value = 1.31×10^−4^), and CCR6 (p-value = 1.56×10^−4^) were the four most significant reporter receptors when considering statistical significance.

**Table 3:** Reporter receptors of cervical cancer (p < 0.05).

**Symbol**	**Name**	**p-value**	**Description**
ABL1	ABL Proto-Oncogene 1	3.88×10^−3^	Involved in a variety of cellular processes, including cell division, adhesion, differentiation, and response to stress.
ATR	ATR Serine/Threonine Kinase	8.88×10^−16^	Phosphorylates checkpoint kinases (CHK1, RAD17 and RAD9) as well as tumor suppressor protein (BRCA1).
CCR6	C-C Motif Chemokine Receptor 6	1.56×10^−4^	Important for B-lineage maturation and antigen-driven B-cell differentiation, and regulate the migration and recruitment of dentritic and T cells during inflammatory and immunological responses.
CD86	T-lymphocyte activation antigen CD86	6.24×10^−3^	Expressed by antigen-presenting cells; the ligand for CD28 antigen and cytotoxic T-lymphocyte-associated protein.
EDNRA	Endothelin Receptor Type A	1.02×10^−2^	G protein-coupled receptor activating a phosphatidylinositol-calcium second messenger system.
EDNRB	Endothelin Receptor Type B	7.38×10^−5^	G protein-coupled receptor activating a phosphatidylinositol-calcium second messenger system.
EGFR	Epidermal Growth Factor Receptor	1.31×10^−4^	Essential for ductal development of the mammary glands.
EPHA4	Ephrin Receptor A4	1.76×10^−2^	Implicated in mediating developmental events, particularly in the nervous system.
EPHA5	Ephrin Receptor A5	3.88×10^−2^	Implicated in mediating developmental events, particularly in the nervous system.
EPHB2	Ephrin Receptor B2	1.16×10^−2^	Previously associated with Prostate Cancer/Brain Cancer Susceptibility, Somatic and Prostate Cancer.
FPR1	Formyl Peptide Receptor 1	2.78×10^−2^	Mediates the response of phagocytic cells to invasion of the host by microorganisms and is important in host defense and inflammation.
GRIK5	Glutamate Ionotropic Receptor Kainate Type Subunit 5	2.45×10^−2^	Previously associated with Schizophrenia.
ITPR1	Inositol 1,4,5-Trisphosphate Receptor Type 1	5.95×10^−3^	Mediates calcium release from the endoplasmic reticulum.
NCOA3	Nuclear Receptor Coactivator 3	2.84×10^−3^	Previously associated with Breast Cancer and Meningothelial Meningioma.
NR2C1	Nuclear receptor subfamily 2 group C member 1	5.21×10^−3^	Function in many biological processes such as development, cellular differentiation and homeostasis.
NR2C2	Nuclear Receptor Subfamily 2 Group C Member 2	1.21×10^−2^	Function in many biological processes such as development, cellular differentiation and homeostasis.
P2RX4	Purinergic Receptor P2X 4	6.05 x10^-12^	A ligand-gated ion channel with high calcium permeability.
RYK	Receptor-Like Tyrosine Kinase	1.41×10^−2^	Previously associated with Robinow Syndrome, Autosomal Dominant 1 and Multiple Endocrine Neoplasia, Type Iib.

### The regulatory codes of cervical cancer

The regulatory elements (TFs or miRNAs) controlling the transcriptional expression of the core genes of cervical cancer were also identified by employing the combinatorial human transcriptional regulatory interaction network. Specifically, three TFs, namely, E2F4 (p-value = 0.013), ETS1 (p-value = 0.014), and CUTL1 (p-value = 0.022), were highlighted (Table 4). We also identified the reporter miRNAs (p-value ≤ 10^−4^) as the key regulatory elements in the transcriptional and post-transcriptional control of the core genes in cervical cancer (Table 5).

**Table 4:** Reporter transcription factors associated with the core genes of cervical cancer (p < 0.05).

Reporter transcription factor	Name	p-value	# of targeted genes	Association of the transcription factor with human diseases
E2F4	E2F Transcription Factor 4	0.013	91	Over-expression was associated with breast and colon cancers; mutation was associated with endometrial, prostate, colorectal, and gastric cancers as well as ulcerative colitis-associated neoplasm; amplification was associated with bladder cancer [47–49].
ETS1	ETS Proto-Oncogene 1	0.014	184	Up-regulation has been linked with cervical, breast and ovarian cancers [50].
CUTL1	Cut Like Homeobox 1	0.022	3	Over-expression was reported in high-grade carcinomas, and cause tubule formation obstruction in breast cancer [51].

**Table 5:** Reporter micro-RNAs associated with the core genes of cervical cancer.

miRNA	p-value	Description
**miR-192-5p**	<10^−15^	Promotes the proliferation and metastasis of hepatocellular carcinoma cell by targeting SEMA3A [52].
**miR-193b-3p**	<10^−15^	Down-regulation was observed in various cancers; over-expression can cause cancer cell proliferation, inhibition, migration and growth [53].
**miR-215-5p**	<10^−15^	Putative tumor suppressor in non-small cell lung cancer [54].
**miR-34a-5p**	8.40×10^−10^	Transcriptional target of p53; decreased expression in several tumors; involved in tumor recurrence inhibition processes [55].
**miR-26b-5p**	3.23×10^−8^	Tumor suppressor; down-regulated in bladder cancer [56].
**miR-92a-3p**	1.56×10^−6^	Over-expression was related to acute myeloid leukemia; associated with colorectal cancer [57–58].
**miR-24-3p**	3.72×10^−6^	Associated with nasopharyngeal carcinoma [59].
**miR-155-5p**	6.17×10^−6^	Behaves as a oncogene or anti-oncogene; asssociated with various diseases including, cancers, viral infections, inflammation and cardiovascular diseases [60].
**miR-484**	9.31×10^−6^	Overexpression was reported in breast cancer, and pancreatic cancer [61].
**miR-26a-5p**	5.41×10^−5^	Down-regulated in colorectal cancer [62].
**miR-1-3p**	5.43×10^−5^	Up-regulation was associated with pregnancy-related complications (i.e. preeclamptic pregnancies) [63].
**miR-124-3p**	6.48×10^−5^	Down-regulation was associated with glioma, oral squamous cell carcinomas, hepatocellular carcinoma and breast cancer [64].
**miR-615-3p**	1.10×10^−4^	Associated with lymphoma and hepatocellular carcinoma [65].
**let-7b-5p**	1.20×10^−4^	Tumor suppressor in multiple myeloma [66].
**miR-93-5p**	1.50×10^−4^	Diagnostic biomarker candidate for primary nasopharyngeal carcinoma [67].
**miR-221-3p**	1.70×10^−4^	Over-expressed in colorectal cancer [68].

### Prognostic power of the reporter biomolecules

**Fig 4:**
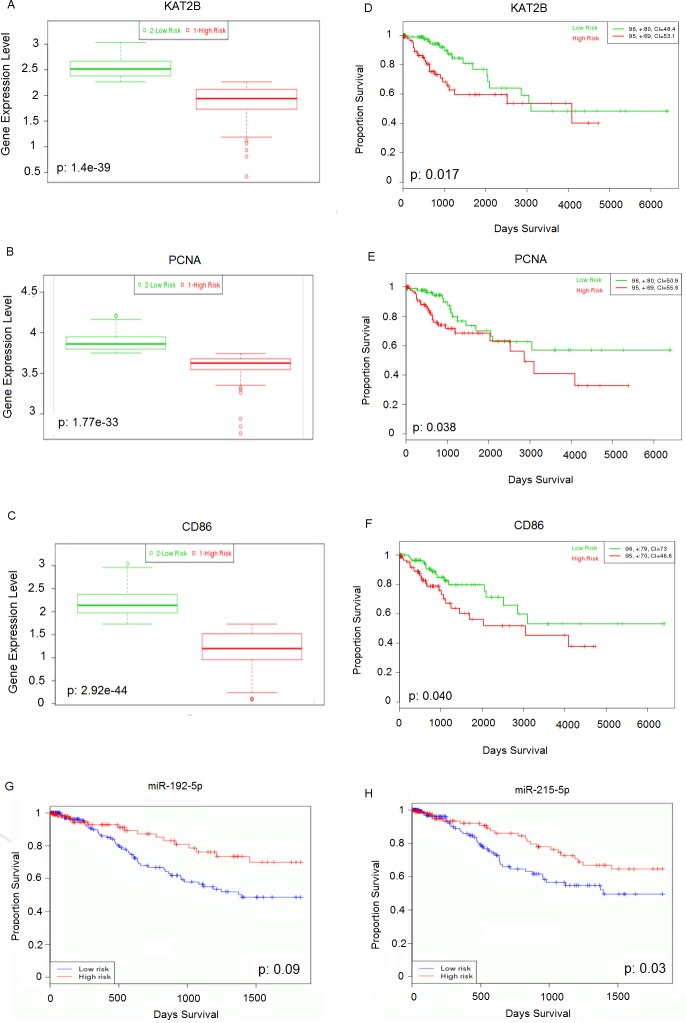
The cross-validation results for reporter biomolecules. Box-plots representing the expression levels of (A) KAT2B, (B) PCNA, and (C) CD86 between the low- and high-risk groups. The Kaplan-Meier curves demonstrating the prognostic power of (D) KAT2B, (E) PCNA, (F) CD86, (G) miR-192-5p, and (H) miR-215-5p. The total size of each group is shown at the top right corner, and the number of censored samples is marked with +.

The validation of differential expression signatures and analysis of the prognostic power of reporter biomolecules (i.e., 10 hubs, 18 receptors, 3 TFs, and 16 miRNAs) were performed using RNA-Seq or miRNA-Seq datasets obtained from independent studies. The samples were partitioned into two groups, namely, low- and high-risk, according to their prognostic performance. The differences in the expression levels of the genes (encoding reporter receptors, TFs, or hub proteins) between the risk groups were represented via box-plots, and prognostic capabilities based on survival data were analyzed by using Kaplan-Meier plots and the log-rank test. The simulations confirmed the significant differences in the expression of all reporter biomolecules between low- and high-risk groups with p-values ranging from 4.75×10^−14^ to 1.47×10^−58^ (S1–S41 Figs). The differential expression profiles and prognostic power of the hub proteins KAT2B (p-value = 0.017, hazard ratio = 2.09) and PCNA (p-value = 0.038, hazard ratio = 1.91), the reporter receptor CD86 (p-value = 0.040, hazard ratio = 1.89), and the reporter miRNAs miR-192-5p (p-value = 0.009) and miR-215-5p (p-value = 0.033) are shown in Fig 4.

**Fig 5:**
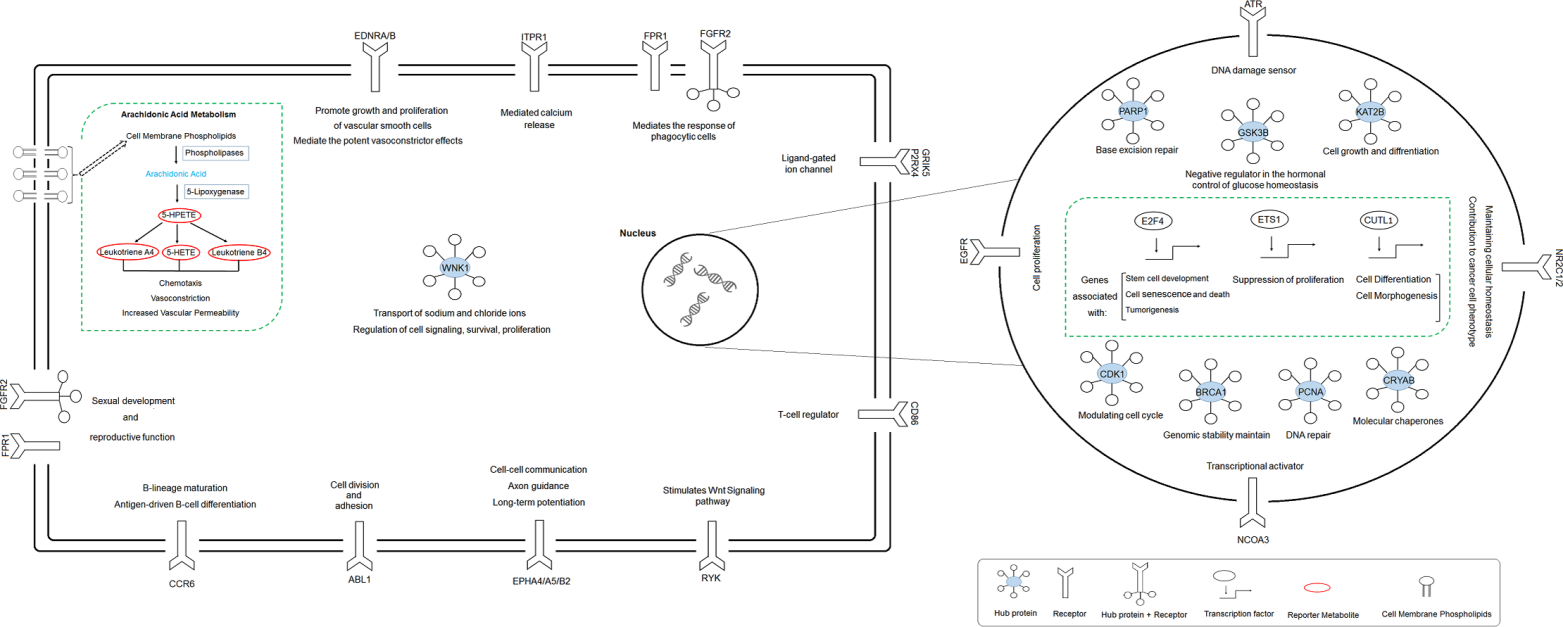
A conceptual summary of reporter biomolecules (receptors, hub proteins, transcription factors, and metabolites) highlighted as potential molecular signatures in cervical cancer.

A conceptual summary of the revealed reporter biomolecules is also depicted (Fig 5).

## Discussion

Considering that oncogenic HPV infection is necessary but not sufficient for the development of cervical cancer, the elucidation of the molecular mechanisms that occur as a consequence of the genetic and environmental factors playing a role in the pathogenesis of this disease is a great challenge. Statistics on the occurrence and high death ratio in cervical cancer reveal the need for novel diagnostic and treatment strategies for cervical cancer. Therefore, the identification of effective prognostic biomarkers and therapeutic targets should be beneficial for increasing the specificity and sensitivity of diagnostic/prognostic tools and for developing novel therapeutics and efficient drug repositioning strategies.

Over the last decade, substantial research has been undertaken to understand the mechanisms of cervical cancer pathogenesis and to identify diagnostic and prognostic targets. However, disease-specific and effective biomarkers remained unavailable because most studies have focused on individual genes associated with cervical cancer and ignored the interactions and associations among the gene products. On the other hand, the systems biology perspective requires the integration of genome-wide biological data with biomolecular networks to elucidate the disease mechanisms and identify the molecular signatures of human diseases [37–38, 69–70]. In this study, we performed a meta-analysis of cervical cancer associated gene expression data, identified the core DEGs of cervical cancer, and integrated this information with comprehensive human biomolecular networks (i.e., PPI, metabolic, and transcriptional regulation) to explore reporter biomolecules that might be useful for developing efficient diagnostic and prognostic strategies in cervical cancer.

On the basis of the individual analysis of gene expression datasets, we observed that hundreds of genes were differentially expressed in each dataset. Moreover, independent of the population considered in sampling (Fig 1), 113 DEGs were down-regulated, and 199 DEGs were up-regulated in all datasets. These core genes of cervical cancer were shown to encode proteins with various molecular functions related to essential biological processes such as the cell cycle, DNA replication, oocyte meiosis, and maturation. The up-regulation of these processes and the p53 signaling pathway could be explained by the rapid proliferation and continuous growth of cancerous cells.

The core genes are associated with a range of human diseases, including various cancers, periodontitis, rheumatoid arthritis, systemic lupus erythematosus, and Alzheimer's disease (Fig 2). The possible association of cervical cancer with these diseases was in accordance with the clinical observations reported in the literature. For instance, the risk of cervical cancer outcome was increased in rheumatoid arthritis [71] and systemic lupus erythematosus [72]. On the other hand, cancer patients were less likely to develop Alzheimer's disease [73]. Furthermore, the periodontal pockets were reported to be reservoirs for several viruses, including HPV [74], thus suggesting the probable association of cervical cancer development and periodontitis, which is a chronic oral infection caused by the synergistic action of some bacteria and viruses. In addition to these diseases, disease pathways for colorectal, bladder, breast, lung, stomach, and prostate cancers were also significantly enriched with the core genes of cervical cancer (p-value < 10^−3^). This may be the result of common molecular mechanisms developed during cancer progression and the response of cells to carcinogenesis.

The reconstruction and topological analysis of PPI networks around the proteins encoded by the core genes of cervical cancer resulted in the identification of hub proteins, that have central roles in the information flow within the networks (Fig 3). BRCA1, ESR1, PCNA, and FGFR2 have already been shown to be associated with cervical cancer [75–78]. Furthermore, the KAT2B, PARP1, CDK1, GSK3B, WNK1, and CRYAB, proteins are associated with various cancer types, but their roles in cervical cancer have not been identified. The tumor suppressor role of the histone acetyltransferase, KAT2B (also known as PCAF or p300/CBP-associated factor) was previously proposed in breast cancer [79], esophageal squamous cell cancer [80], and gastric carcinoma [81]. Furthermore, the interaction of KAT2B with HPV16 E7, which is a cervical cancer associated oncoprotein, was also reported [82]. The chromatin-associated enzyme PARP1, which is one of the core directors of DNA repair and involved in tumorigenesis pathways, was over-expressed in many of the cancer types, such as breast, uterine, ovarian, colorectal, lung, leukemia, and lymphomas at the mRNA and/or protein levels [83]. A significant relationship between Val762Ala polymorphism in PARP-1 and the induced risk of cervical cancer in Caucasian women was also reported [84]. Among the other hub proteins, CDK1, GSK3B, and WNK1 were highlighted. The cyclin-dependent kinases have been reported to be involved in apoptosis, cell division, pain signaling, RNA splicing, neuronal cell physiology, and insulin release processes [85]. CDK1 expression was shown to be up-regulated in lymphoma, advanced melanoma and lung cancer [86]. GSK3 members participate in apoptosis, cell cycle control, insulin action, neuronal cell death, and developmental regulation processes [85] and are associated with various disorders including diabetes, inflammation, neurological, and neoplastic diseases [87]. GSK3B is a main component of the Wnt signaling pathway and may behave like a tumor promoter or suppressor depending on the cancer type. Its action as a tumor promoter was reported in pancreatic, colorectal, stomach, ovarian, thyroid, and prostate cancers, whereas it behaves as a tumor suppressor in oral, esophageal, breast, lung, and skin cancers [87–88]. WNKs are involved in cell proliferation, differentiation, migration, exocytosis regulation, and MAPK/PI3-kinase pathways. WNK1 mutations were previously reported to be associated with breast, ovarian, colorectal, and lung cancers [89]. Moreover, the remaining hub protein, namely, CRYAB, is an alpha crystalline, which acts as a molecular chaperone belonging to the small heat shock protein family. The up-regulation of CRYAB was shown to be associated with several cancers, including renal, breast, thyroid, head and neck, hepatocellular, and nasopharyngeal types [90]. However, the transcriptome datasets utilized here represented CRYAB as being down-regulated in cervical cancer. In terms of the obtained results, the hub proteins KAT2B, PARP1, CDK1, GSK3B, WNK1, and CRYAB have been associated with several cancers in previous studies, but their association with cervical cancer is proposed here for the first time. Furthermore, the differential expression levels of hub proteins between the high- and low-risk groups were cross-validated, and the prognostic power of KAT2B and PCNA was demonstrated in a large RNA-Seq dataset obtained by an independent study (Fig 4). We showed that the down regulation of KAT2B and PCNA expression was associated with a higher risk of cervical cancer. Therefore, these proteins as a whole can be considered systems-level biomarkers that can be used for screening or therapeutic purposes in cervical carcinoma; however, further efforts are needed to confirm the findings at the protein expression level empirically and clinically.

The majority of the core genes of cervical cancer encode enzymes and modulators, thus indicating substantial alterations in cell metabolism during disease progression. Therefore, the reporter metabolites and significantly enriched metabolic pathways around which the most significant transcriptional changes occur were identified (Table 2). Arachidonic acid metabolism was highlighted with 15 reporter metabolites, such as several derivatives of eicosatetraenoic acid, leukotrienes (A4, B4), arachidonate, 5,6-epoxytetraene, and hepoxilin A3. The arachidonic acid pathway regulates inflammatory responses, cell proliferation, survival, invasion and metastasis. Moreover, the activation and the significant roles of the arachidonic acid pathway in carcinogenesis were demonstrated by clinical studies and cell- and animal-based studies. The transformation of arachidonate to hydroperoxyeicosatetraenoic acids (HPETEs), which are subsequently reduced to hydroxyeicosatetraenoic acids (HETEs), is catalyzed by the glutathione peroxidase enzyme by the lipoxygenase pathway. The pro-carcinogenic and anti-carcinogenic roles of HETEs and HPETEs were reported in carcinogenesis [91–92]. Oxoeicosatetraenoic acids (Oxo-ETEs) and hepoxilins are also products of HETEs and were reported to be associated with allergy, asthma, and lung cancer [93–95]. Leukotrienes are effective pro-inflammatory mediators and play key roles in inflammatory diseases, as well as prostate, esophageal, and pancreatic cancers [96]. Therefore, targeting the arachidonic acid pathway for cancer inhibition and/or therapy has become an interesting issue for researchers, and it has been reported that a natural product called “curcumin” has therapeutic potential in cervical cancer by targeting several pathways including the arachidonic acid pathway [97]. PPAR signaling pathway was also significantly enriched with reported metabolites. PPARs are nuclear hormone receptors that are activated by polyunsaturated fatty acids. They can also be activated by arachidonic acid derivatives (i.e., prostaglandins and eicosanoids) [98]. PPARs are used as drug targets to cure metabolic syndrome and type 2 diabetes. They also have a role in cancer cell proliferation [99]. It was established that there is a relationship between cervical cancer and PPAR-gamma, one of the three PPAR subtypes, and that PPAR-gamma can be exploited as a therapeutic target for cervical cancer [100]. Many clinical oncology studies have focused on the association between glutathione metabolism and tumorigenesis; however, the outcomes of these studies were limited and inconsistent. Glutathione levels were found to be elevated in breast and ovarian cancer but reduced in brain and liver tumors. On the other hand, glutathione levels showed inconsistent results in ovarian cancer patients [101].

In this study, the reporter features algorithm was adapted to identify other reporter molecules, namely, receptors, TFs, and miRNAs. Eighteen proteins were identified as reporter receptors (Table 3). Among these receptors, ATR represents a central cellular response regulator that is activated under replication stress and was proposed to be a therapeutic target in cancer therapy [102]. The chemokine receptor CCR6 was reported as a prognostic marker in colorectal cancer because the up-regulation of CCR6 has been associated with colorectal cancer metastasis [103]. Furthermore, a higher expression of CD86 was observed in normal cervical epithelium than in HPV16 positive early cervical intraepithelial lesions [104]. Recently, Tian et al. [105] performed a meta-analysis of systematic data and proposed EGFR up-regulation as a potential prognostic biomarker for cervical cancer. P2RX4 (also known as P2X4) is a member of the purinergic receptors, which were reported to be associated with different cancer types including colorectal, esophageal, prostate, and cervical cancer [106]. Endothelin receptors A and B (EDNRA, and EDNRB) have altered expression levels in multiple cancers, such as colorectal, bladder, prostate, and nasopharyngeal carcinomas, in addition to cervical cancer. The up-regulation of EDNRB was associated with aggressive melanoma, and EDNRB was suggested to be a potential tumor progression biomarker in melanoma [107]. Ephrin, which is also known as erythropoietin-producing human hepatocellular receptor, manages multiple processes that are essential for tissue homeostasis or development, is widely expressed in cancer tissues, and plays a role in the tumor microenvironment. The down-regulation of EPHA4 was previously reported in cervical cancer. However, the association of EPHB2 was not studied in cervical cancer; instead, its down-regulation was reported in colorectal cancer [108]. The nuclear receptor subfamily members NR2C1 and NR2C2 were found to be down-regulated in breast cancer but were not proposed as prognostic markers for any cancer [109]. Consequently, to our knowledge the functional association of receptors including CCR6, EPHB2, NR2C1, and NR2C2 with cervical cancer is being proposed for the first time in this study. The differential expression of CD86, CCR6, EPHB2, NR2C1, and NR2C2 between high- and low-risk groups was also cross-validated here, and the prognostic power of CD86 was demonstrated (Fig 4). We showed that the expression of CD86 is associated with a low-risk of cervical cancer.

The transcriptional expression of the core genes of cervical cancer was controlled by three TFs: E2F4, ETS1, and CUTL1. E2F4 has a crucial role in cell cycle progression and is related to several cancers. E2F4 over-expression was associated with breast and colon cancers; its mutation was associated with endometrial, prostate, colorectal, gastric, and ulcerative colitis-associated neoplasm; and its amplification was associated with bladder cancer [47–49]. The up-regulation of ETS1 has been linked with various types of cancer (e.g., cervical, breast, and ovarian cancers), with a particular association with tumor development and invasion [50]. CUTL1 is involved in cellular proliferation and the cell cycle progression modulating the DNA binding affinity of several kinases. It behaves as either a transcriptional repressor or an activator. Its over-expression was reported in high-grade carcinomas and causes tubule formation obstruction in breast cancer [51]. The targeted genes of these TFs were found to be mainly associated with general biological process terms, such as cellular component organization, cellular process, response to stimulus, metabolic process, and biological regulation. Although the association of cervical cancer with E2F4 and ETS1 was clearly identified, the relationship with CUTL1 was not clearly specified.

With regard to statistical significance, 16 reporter miRNAs were determined. The resultant reporter miRNAs were generally associated with carcinogenesis and acted as oncogenes or anti-oncogenes (Table 5). For instance, miR-192-5p promotes the proliferation and metastasis of hepatocellular carcinoma cells by targeting SEMA3A [52]. The down-regulation of the tumor suppressor miR-193b-3p was observed in various cancers, and its over-expression has been associated with cancer cell proliferation, inhibition, migration, and growth [53]. miR-215-5p was reported to be a putative tumor suppressor in non-small cell lung cancer [54]. miR-34a-5p was determined to be a direct transcriptional target of p53, its expression was decreased in several tumors, and it was proposed as a factor involved in the process of tumor recurrence inhibition [55]. miR-26b-5p behaves as a tumor suppressor, and it was reported that it was down-regulated in bladder cancer [56]. Furthermore, miR-92a-3p over-expression was shown to be related to acute myeloid leukemia [57]. Moreover, miR-92a-3p and miR-24-3p were associated with colorectal cancer [58] and nasopharyngeal carcinoma [59], respectively. miR-155-5p behaves as either an oncogene or an anti-oncogene in carcinogenesis, and miR-155-5p has been associated with various diseases including cancers, viral infections, inflammation, and cardiovascular diseases [60]. Moreover, the expression levels of miR-484, miR-26a-5p, miR-1-3p, miR-124-3p, miR-615-3p, let-7b-5p, miR-93-5p, and miR-221-3p were altered in various disorders including breast cancer, pancreatic cancer, colorectal cancer, pregnancy-related complications, glioma, oral squamous cell carcinomas, hepatocellular carcinoma, lymphoma, nasopharyngeal carcinoma, and multiple myeloma [61–68]. In addition to miR-1-3p, the resultant miRNAs have already been associated with various cancers, but not specifically with cervical cancer. Furthermore, the Kaplan-Meier curves indicated the prognostic value of the miRNAs, miR-192-5p and miR-215-5p (Fig 4). The up-regulation of both miRNAs was associated with high-risk in cervical cancer. Therefore, miR-192-5p and miR-215-5p warrant further mechanistic and functional investigation and have great potential as prognostic biomarkers in cervical cancer.

On the basis of an integrative multi-omics approach, we here present molecular codes of cervical cancer at the RNA (mRNA, miRNA), protein (receptor, TF, enzyme), and metabolite levels (Fig 5). The applied approach identified already-known biomarkers, tumor suppressors, and oncogenes in cervical cancer, as well as novel candidates such as KAT2B, PARP1, CDK1, GSK3B, WNK1, CRYAB, CCR6, EPHB2, NR2C1, NR2C2 and CUTL1. The majority of the genome re-programming was regulated by three transcription factors, namely, E2F4, ETS1, and CUTL1, and 16 miRNAs. Furthermore, the arachidonic acid metabolism pathway was highlighted as a potential therapeutic target. Moreover, the differential expression of all reporter biomolecules between the high- and low-risk groups was cross-validated, and the prognostic power of KAT2B, PCNA, CD86, miR-192-5p, and miR-215-5p was demonstrated. These biological molecules not only represent the association of cervical cancer with biological processes and other diseases, but also have significant potential to be considered systems-level biomarkers that may be used for screening or therapeutic purposes in cervical carcinoma. However, more efforts are required to achieve the experimental and clinical validation of the findings obtained here.

## Supporting information

S1 Table The differentially expressed core genes in cervical cancer. (XLSX)

S1 Fig The prognostic power of BRCA1. The box-plot and Kaplan-Meier curve demonstrating the expression level difference between the low- and high-risk groups and prognostic power for BRCA1 hub respectively. The total size of each group is shown at the top right corner and the number of censoring samples are marked with +.
(DOCX)

S2 Fig The prognostic power of CDK1. The box-plot and Kaplan-Meier curve demonstrating the expression level difference between the low- and high-risk groups and prognostic power for CDK1 hub, respectively. The total size of each group is shown at the top right corner and the number of censoring samples are marked with +.
(DOCX)

S3 Fig The prognostic power of CRYAB. The box-plot and Kaplan-Meier curve demonstrating the expression level difference between the low- and high-risk groups and prognostic power for CRYAB hub, respectively. The total size of each group is shown at the top right corner and the number of censoring samples are marked with +.
(DOCX)

S4 Fig The prognostic power of ESR1. The box-plot and Kaplan-Meier curve demonstrating the expression level difference between the low- and high-risk groups and prognostic power for ESR1 hub, respectively. The total size of each group is shown at the top right corner and the number of censoring samples are marked with +.
(DOCX)

S5 Fig The prognostic power of FGFR2. The box-plot and Kaplan-Meier curve demonstrating the expression level difference between the low- and high-risk groups and prognostic power for FGFR2 hub, respectively. The total size of each group is shown at the top right corner and the number of censoring samples are marked with +.
(DOCX)

S6 Fig The prognostic power of GSK3B. The box-plot and Kaplan-Meier curve demonstrating the expression level difference between the low- and high-risk groups and prognostic power for GSK3B hub, respectively. The total size of each group is shown at the top right corner and the number of censoring samples are marked with +.
(DOCX)

S7 Fig The prognostic power of PARP1. The box-plot and Kaplan-Meier curve demonstrating the expression level difference between the low- and high-risk groups and prognostic power for PARP1 hub, respectively. The total size of each group is shown at the top right corner and the number of censoring samples are marked with +.
(DOCX)

S8 Fig The prognostic power of WNK1. The box-plot and Kaplan-Meier curve demonstrating the expression level difference between the low- and high-risk groups and prognostic power for WNK1 hub, respectively. The total size of each group is shown at the top right corner and the number of censoring samples are marked with +.
(DOCX)

S9 Fig The prognostic power of CUTL1. The box-plot and Kaplan-Meier curve demonstrating the expression level difference between the low- and high-risk groups and prognostic power for reporter transcription factor CUTL1, respectively. The total size of each group is shown at the top right corner and the number of censoring samples are marked with +.
(DOCX)

S10 Fig The prognostic power of E2F4. The box-plot and Kaplan-Meier curve demonstrating the expression level difference between the low- and high-risk groups and prognostic power for reporter transcription factor E2F4, respectively. The total size of each group is shown at the top right corner and the number of censoring samples are marked with +.
(DOCX)

S11 Fig The prognostic power of ETS1. The box-plot and Kaplan-Meier curve demonstrating the expression level difference between the low- and high-risk groups and prognostic power for reporter transcription factor ETS1, respectively. The total size of each group is shown at the top right corner and the number of censoring samples are marked with +.
(DOCX)

S12 Fig The prognostic power of ABL1. The box-plot and Kaplan-Meier curve demonstrating the expression level difference between the low- and high-risk groups and prognostic power for reporter receptor ABL1, respectively. The total size of each group is shown at the top right corner and the number of censoring samples are marked with +.
(DOCX)

S13 Fig The prognostic power of ATR. The box-plot and Kaplan-Meier curve demonstrating the expression level difference between the low- and high-risk groups and prognostic power for reporter receptor ATR, respectively. The total size of each group is shown at the top right corner and the number of censoring samples are marked with +.
(DOCX)

S14 Fig The prognostic power of CCR6. The box-plot and Kaplan-Meier curve demonstrating the expression level difference between the low- and high-risk groups and prognostic power for reporter receptor CCR6, respectively. The total size of each group is shown at the top right corner and the number of censoring samples are marked with +.
(DOCX)

S15 Fig The prognostic power of EDNRA. The box-plot and Kaplan-Meier curve demonstrating the expression level difference between the low- and high-risk groups and prognostic power for reporter receptor EDNRA, respectively. The total size of each group is shown at the top right corner and the number of censoring samples are marked with +.
(DOCX)

S16 Fig The prognostic power of EDNRB. The box-plot and Kaplan-Meier curve demonstrating the expression level difference between the low- and high-risk groups and prognostic power for reporter receptor EDNRB, respectively. The total size of each group is shown at the top right corner and the number of censoring samples are marked with +.
(DOCX)

S17 Fig The prognostic power of EGFR. The box-plot and Kaplan-Meier curve demonstrating the expression level difference between the low- and high-risk groups and prognostic power for reporter receptor EGFR, respectively. The total size of each group is shown at the top right corner and the number of censoring samples are marked with +.
(DOCX)

S18 Fig The prognostic power of EPHA4. The box-plot and Kaplan-Meier curve demonstrating the expression level difference between the low- and high-risk groups and prognostic power for reporter receptor EPHA4, respectively. The total size of each group is shown at the top right corner and the number of censoring samples are marked with +.
(DOCX)

S19 Fig The prognostic power of EPHA5. The box-plot and Kaplan-Meier curve demonstrating the expression level difference between the low- and high-risk groups and prognostic power for reporter receptor EPHA5, respectively. The total size of each group is shown at the top right corner and the number of censoring samples are marked with +.
(DOCX)

S20 Fig The prognostic power of EPHB2. The box-plot and Kaplan-Meier curve demonstrating the expression level difference between the low- and high-risk groups and prognostic power for reporter receptor EPHB2, respectively. The total size of each group is shown at the top right corner and the number of censoring samples are marked with +.
(DOCX)

S21 Fig The prognostic power of FPR1. The box-plot and Kaplan-Meier curve demonstrating the expression level difference between the low- and high-risk groups and prognostic power for reporter receptor FPR1, respectively. The total size of each group is shown at the top right corner and the number of censoring samples are marked with +.
(DOCX)

S22 Fig The prognostic power of GRIK5. The box-plot and Kaplan-Meier curve demonstrating the expression level difference between the low- and high-risk groups and prognostic power for reporter receptor GRIK5, respectively. The total size of each group is shown at the top right corner and the number of censoring samples are marked with +.
(DOCX)

S23 Fig The prognostic power of ITPR1. The box-plot and Kaplan-Meier curve demonstrating the expression level difference between the low- and high-risk groups and prognostic power for reporter receptor ITPR1, respectively. The total size of each group is shown at the top right corner and the number of censoring samples are marked with +.
(DOCX)

S24 Fig The prognostic power of NCOA3. The box-plot and Kaplan-Meier curve demonstrating the expression level difference between the low- and high-risk groups and prognostic power for reporter receptor NCOA3, respectively. The total size of each group is shown at the top right corner and the number of censoring samples are marked with +.
(DOCX)

S25 Fig The prognostic power of NR2C1. The box-plot and Kaplan-Meier curve demonstrating the expression level difference between the low- and high-risk groups and prognostic power for reporter receptor NR2C1, respectively. The total size of each group is shown at the top right corner and the number of censoring samples are marked with +.
(DOCX)

S26 Fig The prognostic power of NR2C2. The box-plot and Kaplan-Meier curve demonstrating the expression level difference between the low- and high-risk groups and prognostic power for reporter receptor NR2C2, respectively. The total size of each group is shown at the top right corner and the number of censoring samples are marked with +.
(DOCX)

S27 Fig The prognostic power of P2RX4. The box-plot and Kaplan-Meier curve demonstrating the expression level difference between the low- and high-risk groups and prognostic power for reporter receptor P2RX4, respectively. The total size of each group is shown at the top right corner and the number of censoring samples are marked with +.
(DOCX)

S28 Fig The prognostic power of RYK. The box-plot and Kaplan-Meier curve demonstrating the expression level difference between the low- and high-risk groups and prognostic power for reporter receptor RYK, respectively. The total size of each group is shown at the top right corner and the number of censoring samples are marked with +.
(DOCX)

S29 Fig The prognostic power of miR-193b-3p. The Kaplan-Meier curve demonstrating the prognostic power of reporter miR-193b-3p.
(DOCX)

S30 Fig The prognostic power of miR-34a-5p. The Kaplan-Meier curve demonstrating the prognostic power of reporter miR-34a-5p.
(DOCX)

S31 Fig The prognostic power of miR-26b-5p. The Kaplan-Meier curve demonstrating the prognostic power of reporter miR-26b-5p.
(DOCX)

S32 Fig The prognostic power of miR-92ab-3p. The Kaplan-Meier curve demonstrating the prognostic power of reporter miR-92ab-3p.
(DOCX)

S33 Fig The prognostic power of miR-24-3p. The Kaplan-Meier curve demonstrating the prognostic power of reporter miR-24-3p.
(DOCX)

S34 Fig The prognostic power of miR-155-5p. The Kaplan-Meier curve demonstrating the prognostic power of reporter miR-155-5p.
(DOCX)

S35 Fig The prognostic power of miR-484. The Kaplan-Meier curve demonstrating the prognostic power of reporter miR-484.
(DOCX)

S36 Fig The prognostic power of miR-26a-5p. The Kaplan-Meier curve demonstrating the prognostic power of reporter miR-26a-5p.
(DOCX)

S37 Fig The prognostic power of miR-124-3p. The Kaplan-Meier curve demonstrating the prognostic power of reporter miR-124-.
(DOCX)

S38 Fig The prognostic power of miR-615-3p. The Kaplan-Meier curve demonstrating the prognostic power of reporter miR-615-3p.
(DOCX)

S39 Fig The prognostic power of let-7b-5p. The Kaplan-Meier curve demonstrating the prognostic power of reporter let-7b-5p.
(DOCX)

S40 Fig The prognostic power of miR-93-5p. The Kaplan-Meier curve demonstrating the prognostic power of reporter miR-93-5p.
(DOCX)

S41 Fig The prognostic power of miR-221-3p. The Kaplan-Meier curve demonstrating the prognostic power of reporter miR-221-3p.
(DOCX)
