# Supplementary material for: Potential biomarkers and therapeutic targets in cervical cancer: Insights from the meta-analysis of transcriptomics data within network biomedicine perspective
Source: PLoS One. 2018 Jul 18;13(7):e0200717. doi: 10.1371/journal.pone.0200717 (PMC6051662; doi:10.1371/journal.pone.0200717)
Supplement: S2 Fig — The box-plot and Kaplan-Meier curve demonstrating the expression level difference between the low- and high-risk groups and prognostic power for CDK1 hub, respectively. The total size of each group is shown at the top right corner and the number of censoring samples are marked with +. (DOCX) [file pone.0200717.s003.docx]

**
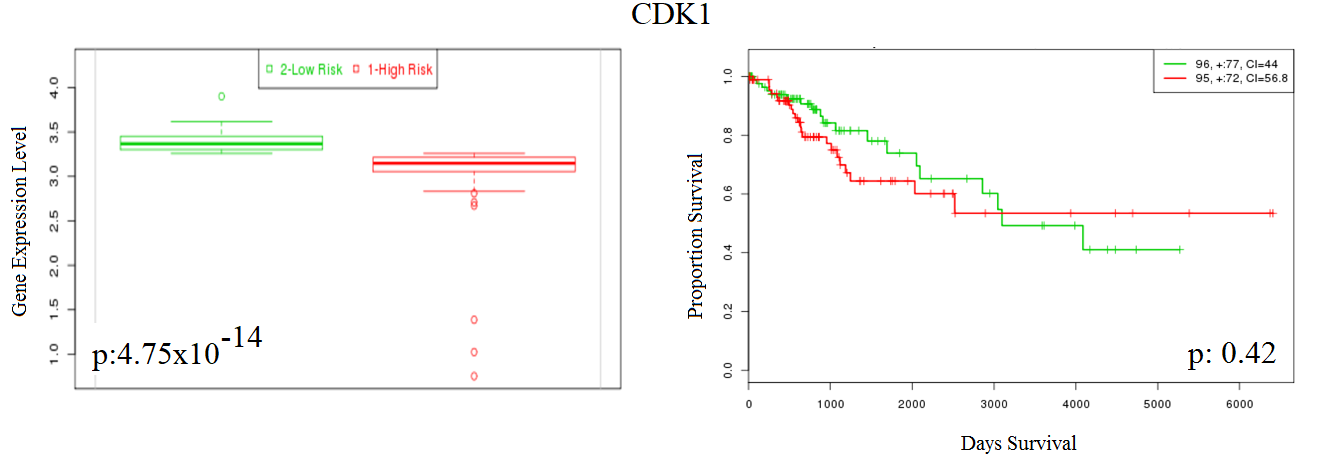
S2 Fig. The prognostic power of CDK1.** The box-plot and Kaplan-Meier curve demonstrating the expression level difference between the low- and high-risk groups and prognostic power for CDK1 hub, respectively. The total size of each group is shown at the top right corner and the number of censoring samples are marked with +.
