# Supplementary material for: Potential biomarkers and therapeutic targets in cervical cancer: Insights from the meta-analysis of transcriptomics data within network biomedicine perspective
Source: PLoS One. 2018 Jul 18;13(7):e0200717. doi: 10.1371/journal.pone.0200717 (PMC6051662; doi:10.1371/journal.pone.0200717)
Supplement: S19 Fig — The box-plot and Kaplan-Meier curve demonstrating the expression level difference between the low- and high-risk groups and prognostic power for reporter receptor EPHA5, respectively. The total size of each group is shown at the top right corner and the number of censoring samples are marked with +. (DOCX) [file pone.0200717.s020.docx]

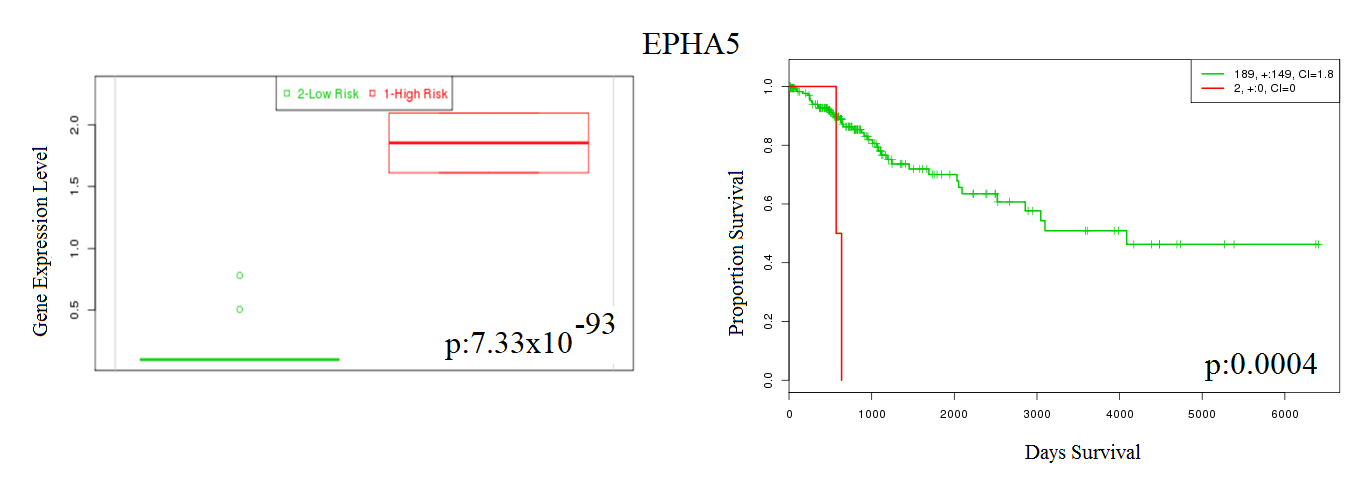
**S19 Fig. The prognostic power of EPHA5.** The box-plot and Kaplan-Meier curve demonstrating the expression level difference between the low- and high-risk groups and prognostic power for reporter receptor EPHA5, respectively. The total size of each group is shown at the top right corner and the number of censoring samples are marked with +.
