# Supplementary material for: Potential biomarkers and therapeutic targets in cervical cancer: Insights from the meta-analysis of transcriptomics data within network biomedicine perspective
Source: PLoS One. 2018 Jul 18;13(7):e0200717. doi: 10.1371/journal.pone.0200717 (PMC6051662; doi:10.1371/journal.pone.0200717)
Supplement: S31 Fig — The Kaplan-Meier curve demonstrating the prognostic power of reporter miR-26b-5p. (DOCX) [file pone.0200717.s032.docx]

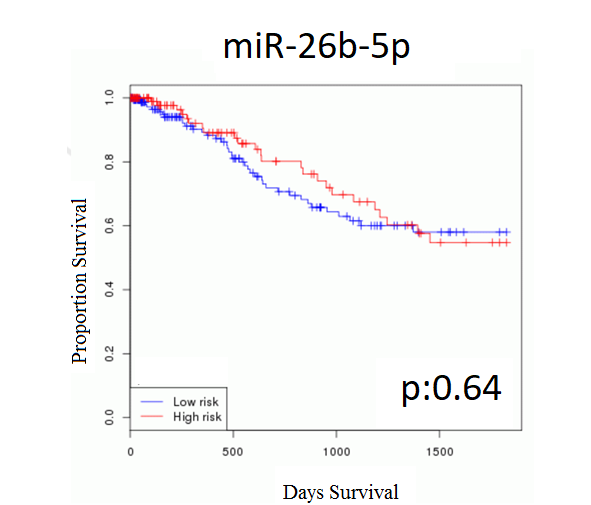
S31 Fig. **The prognostic power of miR-26b-5p**. The Kaplan-Meier curve demonstrating the prognostic power of reporter miR-26b-5p.
