# Supplementary material for: Potential biomarkers and therapeutic targets in cervical cancer: Insights from the meta-analysis of transcriptomics data within network biomedicine perspective
Source: PLoS One. 2018 Jul 18;13(7):e0200717. doi: 10.1371/journal.pone.0200717 (PMC6051662; doi:10.1371/journal.pone.0200717)
Supplement: S35 Fig — The Kaplan-Meier curve demonstrating the prognostic power of reporter miR-484. (DOCX) [file pone.0200717.s036.docx]

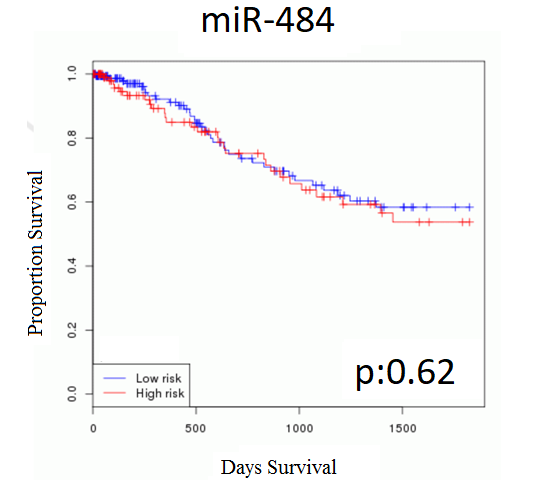
**S35 Fig. The prognostic power of miR-484.** The Kaplan-Meier curve demonstrating the prognostic power of reporter miR-484.
